# Management of Subsyndromal Delirium With Daridorexant and Quetiapine: A Case Report

**DOI:** 10.7759/cureus.85232

**Published:** 2025-06-02

**Authors:** Keitaro Takahashi, Kana Kiryu, Hiroyuki Harada, Tadafumi Kato, Hidetaka Tamune

**Affiliations:** 1 Department of Psychiatry and Behavioral Science, Juntendo University Graduate School of Medicine, Tokyo, JPN

**Keywords:** daridorexant, doras, dual orexin receptor antagonists, morning drowsiness, quetiapine, subsyndromal delirium (ssd)

## Abstract

Subsyndromal delirium (SSD), characterized by subthreshold symptoms of delirium such as disturbances in attention and cognition, is prevalent in the intensive care unit (ICU) and is associated with prolonged hospitalization and delayed functional recovery. However, evidence-based treatment guidelines remain limited. Here, we present a postoperative patient with SSD successfully managed with daridorexant and quetiapine.

A 76-year-old man with a history of pharyngeal cancer and tracheostomy developed acute insomnia in the ICU following surgery for esophageal cancer. Lemborexant was initiated on postoperative day (POD) 6 and titrated to the maximum dose of 10 mg on POD 8, but it caused morning drowsiness that interfered with participation in rehabilitation. On POD 10, he was referred to the Consultation-Liaison team. Based on his disrupted sleep-wake cycle and fluctuating mood following dyspnea due to aspiration pneumonia, he was diagnosed with SSD (Diagnostic and Statistical Manual of Mental Disorders, Fifth Edition, Text Revision (DSM-5-TR)) with comorbid acute insomnia. We initiated daridorexant on POD 10 and increased the dose to 50 mg on POD 12, leading to reduced morning drowsiness and improved sleep initiation. Although these symptoms improved, fluctuating mood and poor appetite persisted slightly. Low-dose quetiapine (12.5 mg) was subsequently added, resulting in stabilization of psychiatric symptoms and steady rehabilitation progress. He was discharged on POD 31, and all psychotropic medications were successfully discontinued at discharge.

Conclusions: Daridorexant, a dual orexin receptor antagonist (DORA) with a shorter half-life, appeared effective in reducing morning drowsiness. As the use of short-acting benzodiazepines has a potential risk of inducing delirium, the combination of daridorexant and low-dose quetiapine may present a therapeutic option for managing SSD with delayed awakening in the postoperative setting.

## Introduction

Subsyndromal delirium (SSD) is a prevalent neuropsychiatric complication in the intensive care unit (ICU) [1]. It is characterized by the presence of some but not all core symptoms of delirium, such as disturbance in attention accompanied by reduced awareness of the environment and additional disturbance in cognition [2]. When the severity of cognitive impairment falls short of that required for the diagnosis of delirium, the patient is diagnosed with SSD [2]. The clinical relevance of SSD is evidenced by its association with prolonged hospitalization, delayed functional recovery, and increased healthcare utilization [1]. Despite its prevalence, evidence-based treatment guidelines remain limited.

ICU patients are particularly vulnerable to SSD due to a convergence of risk factors, including critical illness, polypharmacy, use of sedatives, sleep disruption, and environmental stressors such as noise and lack of natural light. Among these, insomnia is a modifiable risk factor for the development of delirium [2]. The use of conventional hypnotics, including benzodiazepines, is not recommended in the ICU setting due to the potential risk of inducing delirium [3].

Recently, dual orexin receptor antagonists (DORAs), a novel class of sleep-promoting agents, have emerged as a promising therapeutic option. Orexin is a neuropeptide system that regulates arousal and wakefulness; DORAs function by selectively inhibiting orexin-mediated arousal, promoting sleep with a lower risk of delirium. However, the clinical evidence supporting the efficacy of DORAs in the prevention and treatment of delirium, particularly SSD, is still under investigation [4].

Among DORAs, daridorexant is distinguished by its comparatively brief half-life, estimated at ~8 hours, compared to older agents such as suvorexant and lemborexant. This pharmacokinetic profile has the potential to be suitable in critically ill patients by promoting nighttime sleep without causing residual morning sedation. Here, we report a case of a postoperative patient with SSD successfully treated with daridorexant and low-dose quetiapine, indicating a potential pharmacological option for managing SSD with morning drowsiness in the postoperative setting. Written informed consent was obtained from the patient for the publication of this case report. The patient provided consent for the off-label use of quetiapine for SSD. All efforts were made to protect the patient’s privacy.

## Case presentation

A 76-year-old man with a medical history of pharyngeal cancer and tracheostomy was admitted to the intensive care unit (ICU) following robot-assisted thoracoscopic esophagectomy (RATS-E) for esophageal squamous cell carcinoma (post-neoadjuvant stage ypT3N1M0, Stage IIIA). He had no history of psychiatric illness, including alcohol use disorder. Postoperatively, he developed dyspnea due to aspiration pneumonia and difficulty initiating sleep. Lemborexant, a dual orexin receptor antagonist (DORA), was initiated at 5 mg/day on POD 6 and subsequently titrated up to 10 mg/day on POD 8. However, the patient exhibited persistent difficulty initiating sleep and experienced morning drowsiness, which interfered with morning rehabilitation sessions. 

On POD 10, he was referred to the Consultation-Liaison team. His dyspnea resolved, and the transition from the ICU to a general ward was planned. However, he presented with fluctuating mood and a disrupted sleep-wake cycle. His Richmond Agitation-Sedation Scale (RASS) score was -1, and although he passed the auditory attention screening examination, he demonstrated intermittent difficulty sustaining attention. As the disturbances in attention and cognition did not meet the full criteria for delirium, he was diagnosed with SSD, according to the fifth edition of the Diagnostic and Statistical Manual of Mental Disorders Text Revision (DSM-5-TR) [2], with comorbid acute insomnia.

**Figure 1:**
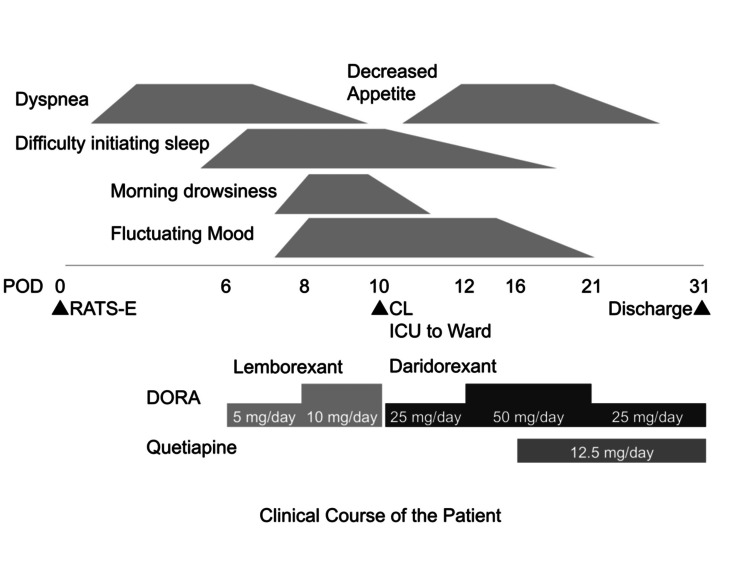
Graphical summary of the clinical course of the patient CL, Consultation-Liaison; DORA, Dual orexin receptor antagonist; POD, Post-operative day; RATS-E, Robot-assisted thoracoscopic esophagectomy.

Lemborexant was discontinued and replaced with daridorexant 25 mg/day, which was increased to 50 mg/day by POD 12. This switch led to a reduction in morning drowsiness and an improvement in sleep initiation. Nevertheless, residual symptoms, including difficulty initiating sleep, fluctuating mood, and decreased appetite, persisted. To address these issues, we introduced low-dose quetiapine (12.5 mg/day) from POD 16, which helped stabilize mood, normalize the sleep-wake cycle, and promote functional recovery. The patient achieved steady rehabilitation progress and was discharged on POD 31, with all psychotropic medications successfully discontinued upon discharge (Figure 1).

## Discussion

Pharmacological characteristics of DORAs and their implications in SSD

Dual orexin receptor antagonists (DORAs) selectively inhibit the orexin system, which plays a key role in maintaining wakefulness [5]. Unlike benzodiazepines, DORAs promote physiological sleep without significantly suppressing REM sleep or causing rebound insomnia upon discontinuation [5]. This pharmacological profile makes DORAs particularly suitable for critically ill patients vulnerable to neurocognitive dysfunction, such as delirium.

In the context of SSD, maintaining a stable sleep-wake cycle is crucial, as disrupted circadian rhythms are a well-established risk factor for delirium [1]. Therefore, hypnotics with minimal side effects are preferred. Indeed, lemborexant was shown to be potentially effective for treating insomnia in cancer patients with delirium in a pilot study [6]. In a multi-center randomized clinical trial, suvorexant significantly reduced hyperactive delirium incidence in older hospitalized adults but did not affect overall delirium rate [4]. The authors suggested that the relatively long half-life of suvorexant may have limited its ability to restore a normal sleep-wake cycle, particularly in hypoactive delirium cases [4]. These findings underscore the potential value of short-acting DORAs for managing critically ill patients with SSD.

Among currently available DORAs, daridorexant is notable for its shorter half-life (~8 hours), which may help minimize morning drowsiness while maintaining the sleep-wake cycle, in contrast to lemborexant and suvorexant, which have longer half-lives (17-19 and ~10 hours, respectively) [5,7] (Table 1).

**Table 1:** Comparison of dual orexin receptor antagonists (DORAs) OX1R: Orexin 1 receptor, OX2R: Orexin 2 receptor, Tmax: Time to maximum concentration, T1/2: Half life. ^†^OX1R/OX2R inhibition, Tmax, and T1/2 data were from interview forms of each drug and Keks et al. [7]

	Daridorexant	Lemborexant	Suvorexant
OX1R inhibition^†^	0.47 nM (Kb)	8.1 nM (Ki)	0.55 nM (Ki)
OX2R inhibition^†^	0.93 nM (Kb)	0.48 nM (Ki)	0.35 nM (Ki)
Tmax^†^	0.8-1.2 h	1.0~1.5 h	~1.5 h
T_1/2_^†^	~8 h	17-19 h	~10 h
Main target	Reduce wakefulness (OX1R)		Reduce wakefulness (OX1R)
	+ Sleep maintenance (OX2R)	Sleep maintenance (OX2R)	+ Sleep maintenance (OX2R)

In the present case, initial treatment with lemborexant improved nighttime awakenings but was accompanied by morning drowsiness [6]. Despite clinical improvements, including relief from dyspnea and transition from the ICU, SSD symptoms persisted and interfered with participation in rehabilitation. Therefore, we considered switching to daridorexant as a potentially beneficial intervention. This switch was associated with reduced morning drowsiness and improved sleep initiation, supporting the importance of selecting hypnotics based on pharmacokinetic profiles individualized to the patient’s condition.

Role of low-dose quetiapine as adjunctive therapy

Despite the improvements in morning alertness, residual sleep initiation difficulty and affective symptoms necessitated adjunctive therapy. Low-dose quetiapine (12.5 mg) was selected for its potent antihistaminic effect at minimal doses, promoting sleep onset without significant dopamine antagonism or antipsychotic burden. Low-dose quetiapine has been used to address refractory insomnia and delirium in ICU settings [8-10]. Although concerns exist regarding metabolic and cardiovascular side effects at higher doses, short-term use at minimal doses is generally well tolerated [11,12]. In this case, quetiapine augmentation led to rapid improvement in mood and appetite stabilization. Consequently, rehabilitation and discharge were facilitated without adverse events, and all psychotropic medications were successfully discontinued upon discharge. When quetiapine is contraindicated, alternative antipsychotics such as risperidone, perospirone, aripiprazole, and brexpiprazole may be considered, although robust evidence supporting their efficacy in this context is currently lacking.

Limitations and future perspectives

Due to the single-case design and the absence of objective assessments of sleep or cognition, causal relationships between medication changes and clinical improvement cannot be definitively established. For example, the stabilization of psychiatric symptoms may have been influenced by the improvement of pneumonia, the transition from the ICU to a general ward, or changes in the environment or nursing care. Adequately sized randomized studies are required to establish the efficacy of pharmacological intervention in the prevention and treatment of SSD and delirium. Future trials with DORAs should focus on delirium subtypes and dimensional outcomes to promote delirium-informed care [13]. In addition, combination strategies, using DORAs for sleep-wake cycle stabilization alongside antipsychotics or another class of psychotropics, may represent a promising approach for critically ill patients with SSD or delirium.

## Conclusions

In this postoperative patient with SSD, switching from lemborexant to daridorexant was associated with increased morning alertness. The shorter half-life of daridorexant likely contributed to the restoration of the sleep-wake cycle without causing residual sedation. Augmentation with low-dose quetiapine further alleviated psychiatric symptoms, thereby facilitating participation in rehabilitation and enabling discharge. Given the limitations of benzodiazepines and the need to preserve daytime function, a combination of appropriate DORAs and low-dose antipsychotics may represent a promising therapeutic option for managing SSD with delayed awakening. Further research is warranted to validate this approach.
